# Generative modelling meets Bayesian inference: a new paradigm for inverse problems

**DOI:** 10.1098/rsta.2024.0334

**Published:** 2025-06-19

**Authors:** Alain Oliviero-Durmus, Yazid Janati, Eric Moulines, Marcelo Pereyra, Sebastian Reich

**Affiliations:** ^1^ Centre de Mathématiques Appliquées, Ecole Polytechnique, Palaiseau, Île-de-France, France; ^2^ Ecole Polytechnique, Palaiseau, Île-de-France, France; ^3^ Heriot–Watt University, Edinburgh, UK; ^4^ University of Potsdam, Potsdam, Germany

**Keywords:** modelling, inverse problems, Bayesian

## Abstract

This special issue addresses Bayesian inverse problems using data-driven priors derived from deep generative models (DGMs) and the convergence of generative modelling techniques and Bayesian inference methods. Conventional Bayesian priors often fail to accurately capture the properties and the underlying geometry of complex, real-world data distributions. In contrast, deep generative models (DGMs), which include generative adversarial networks (GANs), variational auto-encoders (VAEs), normalizing flows and diffusion models (DMs), have demonstrated tremendous success in capturing detailed data representations learned directly from empirical observations. As a result, these models produce priors endowed with superior accuracy, increased perceptual realism and enhanced capacities for uncertainty quantification within inverse problem contexts. This paradigm emerged in the late 2010s, when pioneering efforts were made to explicitly formulate Bayesian inverse problems using conditional Wasserstein generative adversarial networks (GANs). These advances have greatly improved methods for quantifying uncertainties, especially in large-scale imaging applications. Building on these fundamental insights, posterior sampling techniques utilizing DMs have demonstrated remarkable efficiency and robustness, highlighting their potential to effectively tackle complex and diverse inverse problems. The articles collected herein provide essential theoretical breakthroughs and significant algorithmic innovations, collectively demonstrating how deep generative priors mitigate traditional limitations and profoundly enrich both the practical applicability and theoretical foundations of Bayesian inversion.

This article is part of the theme issue ‘Generative modelling meets Bayesian inference: a new paradigm for inverse problems’.

## 1. Introduction

This special issue is devoted to Bayesian inverse problems for which the prior is defined in a data-driven manner. Below, we first outline the general problem setting before summarizing the specific contribution contained in this volume.

Broadly speaking, we consider statistical estimation problems involving an unknown signal of interest x and some indirect or noisy observations y , related to x by a so-called forward mathematical model describing the data acquisition process [1]. Let us formally consider a forward observation model of the form y=G(x)+η , where G maps the parameter space to the observation space and η stands for measurement noise. We are particularly interested in problems where the estimation of x from y is ill-posed or ill-conditioned, typically when this problem does not admit a unique solution or there exists a unique solution but it is not stable with respect to small perturbations in y . Such problems are referred to as inverse problems. They are frequently encountered across various fields of science and engineering and have long been a central focus of research in the mathematical sciences.

Inverse problems can be successfully tackled by leveraging additional information about the unknown signal x to regularize the estimation problem, as well as to reduce uncertainty about x given the observed data y . Following this rationale, the most common approach is to search for a solution $\hat{x}$ that minimizes a composite objective:


$$\hat{x}=\arg\min_{x}\;\mathrm{Loss}(y,G(x))+\lambda \;\mathrm{Pen}(x),$$

where Loss⁡ is a loss function that measures data fidelity and Pen⁡ is a penalization or regularization function encoding expected or desired properties on x . Such formulations have a very long history that can be traced back to the early work of Tikhonov [2]. By choosing the penalization Pen⁡ appropriately, e.g. by tuning the hyperparameter λ , one can favour solutions with desired properties, such as smoothness of the solution, sparsity in some appropriately chosen basis of functions, obtain a well-posed optimization, etc. This includes classical Tikhonov (ridge) regularization and sparsity-promoting penalties, e.g. total variation or sparse decomposition in Fourier or wavelet bases [2–4].

Alternatively, the Bayesian statistical paradigm offers a powerful mathematical and decision-theoretic framework for performing inference on x . This framework relies on statistical models to represent the data observation process and the prior information available. Bayesian decision theory then allows deriving estimators for x and quantifying the uncertainty in the solutions delivered, as well as performing a range of other sophisticated inferences, e.g. hypothesis tests, model self-calibration, model comparison and prediction. We refer the reader to [5–7] for excellent introductions to the topic.

In the Bayesian approach to inverse problems, the signal x is treated as a random variable. Information that reflects the knowledge about the signal x before seeing y is introduced into the problem by specifying the marginal distribution of x , referred to as the *prior* distribution. We write informally the prior as a probability density function (p.d.f.) prior⁡(x) with respect to some reference measure. A rigorous treatment of the prior is beyond our current scope, as well as extending Bayesian inference into a functional framework that demands a significantly more careful and precise definition of p.d.f. and reference measures; see, e.g. Lassas *et al*. [8] and Stuart [7].

The forward model and the noise distribution determine the likelihood function of the observation y , x↦likelihood⁡(y|x) , which is formally the p.d.f. of the conditional distribution of the observation y given the signal x . Bayes’ rule then provides the *posterior* p.d.f.


posterior⁡(x|y)∝likelihood⁡(y|x)×prior⁡(x),

which models our updated knowledge about x after observing y . Many classical inversion methods can be interpreted in this framework: for example, when x and y are both random vectors and assuming a Gaussian prior, a Gaussian likelihood and a linear forward model lead to a Gaussian posterior whose mean and mode (maximum a posteriori) estimator corresponds to the Tikhonov solution. However, the complete characterization of the posterior for large problems is extremely challenging. Sampling-based inference (Markov chain Monte Carlo (MCMC)) or variational approximations are possible in principle, but are often prohibitively expensive when x has a very high dimension, e.g. millions of unknowns in a two-dimensional/three-dimensional image; see, e.g. Cotter *et al*. [9], Bardsley *et al*. [10] and Beskos *et al*. [11].

A crucial part of Bayesian inversion is the choice of the prior. Common priors include simple generic models such as Gaussian random fields, which yield a quadratic regularization, or Markov random fields, which enforce a more complex form of smoothing. Although these analytical priors are mathematically convenient and commonly used, they often do not do justice to the true complexity of the signals of interest. For example, natural images or geophysical fields exhibit rich non-Gaussian structures that cannot be adequately represented by general smoothness or sparsity priors [12–14].

This limitation has sparked interest in more statistically meaningful *data-driven* priors. Deep learning has opened up new avenues for creating meaningful prior models in recent years. A generative model trained on a representative dataset of examples for x , e.g. images of a specific class, can generate new examples that resemble the training data. Such models thus provide a powerful means of specifying prior⁡(x) in a learned, non-parametric way, rather than relying on hand-crafted mathematical formulas. *Deep generative models* (DGMs), which include generative adversarial networks (GANs), variational auto-encoders (VAEs), normalizing flows and more recently diffusion models (DMs), have shown a remarkable ability to learn complex high-dimensional distributions directly from many different types of data. The literature on generative models is extremely vast, as this area of artificial intelligence has witnessed the most spectacular developments over the past decade; see, e.g. Goodfellow *et al*. [15], Rezende & Mohamed [16], Kingma *et al*. [17] and Ho *et al*. [18].

Generative models used as priors are inherently adapted to the distributional complexity of the underlying data, and therefore provide reconstructions of greater accuracy and perceptual realism compared to classical hand-crafted priors. In addition, this innovative approach significantly improves the quantification of uncertainty and provides clearer insights into the variability and reliability of the reconstructions. The use of Bayesian samplers based on DGMs also allows practitioners to obtain multiple plausible solutions from the posterior distribution, effectively mapping the entire landscape of possible reconstructions.

Although it is difficult to determine the exact origins of this methodology, a substantial body of foundational work has emerged between 2016 and 2020. In particular, Bora *et al*. [19] pioneered the explicit use of deep generative priors for ill-posed inverse problems by introducing the compressed sensing with generative models (CSGM) framework. This groundbreaking work showed empirically and theoretically that images lying in the latent space of pre-trained GANs can be reliably reconstructed from significantly fewer measurements than required by traditional sparsity-based methods, setting a strong precedent for generative priors.

Ulyanov *et al*. [20] expanded the conceptual landscape by proposing the deep image prior, which exploits the intrinsic inductive bias of untrained convolutional neural networks. Remarkably, their approach underscored that a randomly initialized network structure itself is sufficient to capture rich image priors that enable effective reconstruction tasks such as denoising, super-resolution and inpainting without explicit prior training.

Another significant advance has been provided by Adler & Öktem [21], who were the first to rigorously formulate Bayesian inverse problems, explicitly using DGMs to quantify uncertainties. Their novel methods, including deep posterior sampling via conditional Wasserstein GANs and direct Bayesian estimation by neural networks, have demonstrated practical Bayesian inversion in large-scale scenarios such as three-dimensional computed tomography, providing posterior means and variances with unprecedented computational efficiency.

Jalal *et al*. [22] introduced score-based generative models, in particular DMs, into Bayesian inversion frameworks. They convincingly illustrated how DMs trained to estimate gradients of logarithmic data density can robustly solve inverse problems such as magnetic resonance imaging (MRI) reconstruction. By integrating Langevin dynamics for posterior sampling into the CSGM framework, they demonstrated reconstructions that are competitive with supervised learning approaches and remain robust in the presence of distributional changes.

We do not claim here to be exhaustive in our citations, as numerous studies have emerged on this topic; rather, these references serve to illustrate the major trends that appeared shortly after the significant emergence of generative models in the late 2010s and have since influenced ongoing research.

Recently, research has developed methods and theories for incorporating generative models into inversion and demonstrated their effectiveness in applications ranging from biomedical imaging to seismic and astronomical inference. In the remainder of this special issue, we provide an overview of these developments. We review the formulation and theoretical foundations of Bayesian inversion problems with learned priors, discuss how they are used in this setting, highlight state-of-the-art algorithms for computing posteriors and outline open challenges for future research.

Despite these promising results, the use of DGMs in Bayesian inversion brings new challenges. A learned generative prior typically concentrates probability mass in a low-dimensional manifold of X , so any true solution outside the range of G cannot be represented, leading to biased recovery. Computational cost is another concern: inference with a deep prior requires repeated passes through a potentially expensive neural network and solving a generally non-convex optimization or sampling problem. However, ongoing algorithm improvements, e.g. more efficient gradient-based samplers, and hardware acceleration gradually mitigate these costs.

## 2. Overview of the survey

We now provide a brief introduction to the 12 contributions collected in this special volume while also putting them into the overarching perspective of Bayesian inference with generative priors.

In ‘Conditional sampling within generative diffusion models’, Zhao *et al*. review computational strategies for conditional sampling using generative DMs. The authors critically examine approaches that overcome the lack of explicit conditional sampling by using joint or marginal distributions with explicit likelihoods. They present and detail several methods, including joint bridging methods that utilize joint distributions and forward–backward path bridging methods formulated as stochastic filtering problems. The paper further explores the promising approach of conditional generative Feynman–Kac models and demonstrates their potential to integrate approximate likelihood information with pre-trained generative models efficiently. A pedagogical example clearly illustrates these methods and highlights practical implementation considerations and performance comparisons with traditional MCMC approaches. This contribution offers a clear exposition of conditional sampling and helps in selecting methods based on application-specific requirements.

In ‘Bridging Diffusion Posterior Sampling and Monte Carlo methods: a survey’, Janati *et al*. provide a systematic overview of methods that combine DMs with Monte Carlo sampling techniques for inverse Bayesian problems. The authors focus on using pre-trained denoising DMs as powerful data-driven priors that enable posterior sampling without re-training. They outline how current methods address the primary challenge, that is, steering DMs towards posterior distributions, through innovative *twisting* mechanisms and gradient-based steering techniques. Various Monte Carlo strategies, including sequential Monte Carlo methods, Gibbs sampling, Langevin dynamics and variational inference (VI), are critically discussed regarding their suitability, strengths and computational costs. Particular attention is given to methods such as diffusion posterior sampling, pseudo-inverse guided diffusion model, Monte Carlo diffusion and midpoint posterior guidance sampling, analysing their practical efficiency, computational trade-offs and scalability.

In ‘Bayesian computation with generative diffusion models by Multilevel Monte Carlo’, Haji-Ali *et al*. address the computational challenges posed by DMs used in Bayesian inverse problems, particularly in computational imaging. Although DMs offer state-of-the-art performance by sampling highly accurate posterior distributions, their computational cost remains prohibitive due to numerous neural function evaluations (NFEs). The authors propose a novel multilevel Monte Carlo (MLMC) strategy to tackle this challenge. By coupling DMs at varying accuracy levels, MLMC efficiently balances computational cost and accuracy. Their method leverages the hierarchical nature of DMs, significantly reducing the overall number of required NFEs without compromising accuracy. Demonstrated across three standard imaging tasks, namely, image denoising, super-resolution and inpainting, the approach achieves a remarkable computational efficiency gain, reducing computational costs by a factor of 4–8 compared to conventional methods. This advancement significantly enhances the practicality of DMs in large-scale Bayesian computations, especially for uncertainty quantification tasks, enabling broader application in science and engineering where computational resources are limited while high accuracy is indispensable.

In ‘Generative diffusion models in infinite dimensions: a survey’, Franzese and Michiardi give an overview of diffusion-based generative models extended to infinite-dimensional Hilbert spaces, which is essential for applications with continuous or very high-resolution data. These DMs, which are typically studied in finite dimensions, are explored in an infinite-dimensional framework through stochastic differential equations (SDEs). The paper reviews the theoretical foundations, including SDEs in Hilbert spaces, and highlights the mathematical challenges and conditions required for the generalization of DMs. Various generative techniques such as time-reversed diffusion processes, annealed Langevin dynamics and functional flow matching are covered. In addition, the authors examine relevant neural architectures suitable for modelling functional spaces and discuss critical training strategies, focusing on resolution invariance and computational efficiency. The review also explores applications of these models to solving Bayesian inverse problems, stochastic optimal control and conditional generation of functional data. The authors emphasize the significant potential of infinite-dimensional generative DMs to advance computational methods in physics-informed machine learning, uncertainty quantification and data assimilation in various scientific and engineering disciplines.

In ‘Stable generative modeling using Schrödinger bridges’, Gottwald *et al*. introduce a novel generative modelling framework that effectively integrates Schrödinger bridges with Langevin dynamics to address stability issues commonly encountered in high-dimensional inverse problems. The authors propose approximating conditional transition probabilities directly from available data via Schrödinger bridges, bypassing the explicit estimation of score functions. By aligning the kernel bandwidth with the discrete time step of a reversible Langevin sampler, their method achieves unconditional stability, circumventing limitations of standard discretization schemes. Furthermore, the paper introduces an innovative split-step scheme ensuring generated samples consistently remain within the convex hull of training data, significantly enhancing numerical robustness. Demonstrating applications on synthetic data, stochastic subgrid-scale parametrization and trajectory generation for dynamical systems, the study validates the accuracy and stability improvements provided by this method. The approach promises broader applicability in fields requiring reliable Bayesian inference and conditional sampling, notably where traditional methods struggle due to instability and high computational demands. This work advances generative modelling techniques, particularly in complex scenarios involving high-dimensional and nonlinear data distributions.

In ‘Bayesian inference for geophysical fluid dynamics using generative models’, Lobbe *et al*. present a method that leverages generative DMs to enhance data assimilation in complex, high-dimensional fluid dynamic problems. Addressing the challenging calibration and uncertainty quantification inherent in geophysical fluid dynamics, the authors employ a diffusion-based generative model, specifically the diffusion Schrödinger bridge, to produce synthetic data that statistically match observed numerical solutions. This approach significantly reduces computational complexity by effectively assimilating data from a finely resolved rotating shallow water system into a lower-dimensional stochastic approximation. The synthetic data are integrated into a particle filtering framework enhanced by tempering and jittering, successfully managing complex, multi-modal posterior distributions. The results demonstrate substantial improvements in accuracy and efficiency, validating generative models as powerful tools for pre-filter calibration. By significantly reducing the computational burden while maintaining predictive accuracy, this methodology has broad implications for real-world applications in meteorology, oceanography and climate modelling, where efficient and precise data assimilation is critical.

In ‘A primer on variational inference for physics-informed deep generative modeling’, Glyn-Davies *et al*. provide a comprehensive overview of VI, which was developed specifically for physics-based problems involving partial differential equations. The article highlights the effectiveness of VI as a scalable and computationally efficient Bayesian inference technique that is well suited for addressing the inherent uncertainties and complexities in inverse modelling scenarios. The authors discuss how VI can seamlessly integrate into DGMs to overcome forward and backward physical inference challenges. They explore several recent advances that illustrate the flexibility of VI and its ability to incorporate physically informed constraints directly into learning systems. The review covers significant variants, including physics-informed neural networks, normalizing flows and VAEs. It highlights their applicability in diverse fields such as geophysics, computational imaging and medical diagnostics. It improves the predictive capabilities and quantification of uncertainties in complex physical systems.

In ‘Taming diffusion models for image restoration: a review’, Luo *et al*. provide an overview of recent advances and methods for using DMs for image restoration tasks. The authors emphasize the strengths of DMs, especially their stability during training and their effectiveness in generating highly realistic images. This overcomes the limitations of conventional image restoration methods, which rely heavily on task-specific expertise. The paper describes various conditional DMs explicitly tailored to image restoration, including direct conditional diffusion, training-free approaches, and advanced techniques such as diffusion bridges and Schrödinger bridges. The authors address current challenges, such as dealing with data that are not distributed and improving the consistency of the generated images. At the same time, they suggest promising directions, including the incorporation of vision language models and optimal transport methods. The paper bridges the gap between theoretical advances and practical implications in computational image and signal processing.

In ‘Denoising: a powerful building-block for imaging, inverse problems, and machine learning’, Milanfar and Delbracio emphasize the fundamental role of denoising. In the past, denoising was mainly considered as noise reduction. Today, it is an essential process that supports more complex tasks such as inverse problems and generative modelling. The authors highlight the structural properties that define effective denoising methods and emphasize their theoretical and practical features, and in particular their identity-preserving, conservative nature and affine combinatorial closure. The paper provides a unified framework that combines the traditional Bayesian and modern machine learning perspectives and presents denoising as a gradient descent step on an implicit energy landscape. Furthermore, it illustrates how denoisers can effectively encode data priors, which significantly enhances the solution of inverse problems through frameworks such as regularization by denoising. Finally, the authors combine classical denoising with contemporary diffusion-based generative models, explaining their operating principles and applicability to posterior sampling in inverse problems. This synthesis emphasizes the broad applicability and essential role of denoising. It highlights the transition from a classical image restoration technique to a central, versatile tool for computational image processing, signal processing and advanced machine learning methods.

In ‘Generative priors for MRI reconstruction trained from magnitude-only images using phase augmentation’, Luo *et al*. present a novel method to construct robust generative image priors specifically for MRI reconstruction. Traditionally, MRI reconstruction techniques that utilize generative models rely on images with complex values containing size and phase information. However, obtaining datasets with complete complex data can be challenging. To address this problem, the authors present a phase augmentation workflow in which realistic phase information is synthetically generated and added to readily available MR image datasets containing only magnitude information. Using these augmented datasets, generative models trained to predict images with complex values significantly improve reconstruction quality compared to models trained for size only. The authors demonstrate the superior performance of these generative priors over traditional $\ell_{1}$ wavelet regularization, especially in highly undersampled, parallel compressed sensing imaging scenarios. Their approach leverages large, existing scale-only databases to produce robust generative priors that significantly improve reconstruction quality and reliability. The results of the study illustrate the critical impact of incorporating synthetic phase data, expanding practical applicability in clinical and research environments and promoting efficient, high-quality MRI without additional complex data acquisition.

In ‘Deep generative models for Bayesian inference on high-rate sensor data: applications in automotive radar and medical imaging’, Stevens *et al*. explore advanced DGMs as Bayesian priors for challenging inverse problems in sensor data processing. Traditionally used for natural image restoration, DGMs face unique difficulties when applied to raw sensor data from radar and medical imaging due to high dynamic range, complex interference and rapid acquisition rates. To overcome these challenges, the authors propose innovative methods for modelling structured noise explicitly using diffusion-based DGMs, significantly improving robustness in scenarios with complex, non-Gaussian interference. The paper further addresses practical constraints by introducing techniques such as active compressed sensing and temporal inference strategies that drastically reduce computational latency, making real-time Bayesian inference feasible. The study demonstrates substantial gains in imaging accuracy and signal reconstruction quality through illustrative applications in automotive radar interference mitigation and medical ultrasound multipath scattering. These contributions extend the applicability of deep generative priors to real-world sensing technologies, paving the way for significant advancements in robust, real-time decision-making systems across multiple engineering and medical domains.

In ‘Reconstructing ECG from indirect signals: a denoising diffusion approach’, Bedin *et al*. present RhythmDiff, an innovative diffusion-based generative model explicitly tailored to reconstruct 12-lead electrocardiogram (ECG) signals with high accuracy. By integrating structured state-space modelling with Bayesian inference, RhythmDiff efficiently overcomes critical challenges in ECG analysis, such as noise reduction, artefact management and reconstruction of missing leads. The authors utilized RhythmDiff as a Bayesian prior. They developed the midpoint guidance posterior sampling (MGPS) algorithm, which enables robust conditional ECG reconstruction under different noise conditions and varying degrees of data completeness. Extensive evaluations show that RhythmDiff and MGPS perform better than existing state-of-the-art methods on several tasks, including reconstructing missing leads and removing artefacts. These advances significantly improve the clinical utility of ECG data, especially in resource-constrained environments and wearable technologies. By improving the fidelity and reliability of reconstructed signals, the proposed method promotes broader and more accessible cardiac health monitoring.

## Data Availability

This article has no additional data.
